# A Prospective Multicenter Trial to Evaluate Urinary Metabolomics for Non-invasive Detection of Renal Allograft Rejection (PARASOL): Study Protocol and Patient Recruitment

**DOI:** 10.3389/fmed.2021.780585

**Published:** 2022-01-07

**Authors:** Miriam C. Banas, Georg A. Böhmig, Ondrej Viklicky, Lionel P. Rostaing, Thomas Jouve, Lluis Guirado, Carme Facundo, Oriol Bestard, Hermann-Josef Gröne, Kazuhiro Kobayashi, Vladimir Hanzal, Franz Josef Putz, Daniel Zecher, Tobias Bergler, Sindy Neumann, Victoria Rothe, Amauri G. Schwäble Santamaria, Eric Schiffer, Bernhard Banas

**Affiliations:** ^1^Department of Nephrology, University Hospital Regensburg, Regensburg, Germany; ^2^Division of Nephrology and Dialysis, Department of Medicine III, Medical University of Vienna, Vienna, Austria; ^3^Transplant Laboratory, Institute for Clinical and Experimental Medicine (IKEM), Prague, Czechia; ^4^Department of Nephrology, Institute for Clinical and Experimental Medicine (IKEM), Prague, Czechia; ^5^Nephrology, Hemodialysis, Apheresis and Kidney Transplantation Department, Grenoble University Hospital, Grenoble, France; ^6^Faculty of Health, Grenoble Alpes University, Grenoble, France; ^7^Nephrology Department, Fundació Puigvert, Instituto de Investigaciones Biomédicas Sant Pau (IIB-Sant Pau), Medicine Department-Universitat Autónoma de Barcelona, REDinREN, Instituto de Investigación Carlos III, Barcelona, Spain; ^8^Vall d'Hebron University Hospital (HUVH), Vall d'Hebron Research Institute (VHIR), Barcelona, Spain; ^9^Institute of Pharmacology, Philipps-University, Marburg, Germany; ^10^Institute of Pharmacology, University of Marburg, Marburg, Germany; ^11^numares AG, Regensburg, Germany

**Keywords:** kidney transplant rejection, urinary metabolites, biomarker, NMR-spectroscopy, non-invasive test

## Abstract

**Background:** In an earlier monocentric study, we have developed a novel non-invasive test system for the prediction of renal allograft rejection, based on the detection of a specific urine metabolite constellation. To further validate our results in a large real-world patient cohort, we designed a multicentric observational prospective study (PARASOL) including six independent European transplant centers. This article describes the study protocol and characteristics of recruited better patients as subjects.

**Methods:** Within the PARASOL study, urine samples were taken from renal transplant recipients when kidney biopsies were performed. According to the Banff classification, urine samples were assigned to a case group (renal allograft rejection), a control group (normal renal histology), or an additional group (kidney damage other than rejection).

**Results:** Between June 2017 and March 2020, 972 transplant recipients were included in the trial (1,230 urine samples and matched biopsies, respectively). Overall, 237 samples (19.3%) were assigned to the case group, 541 (44.0%) to the control group, and 452 (36.7%) samples to the additional group. About 65.9% were obtained from male patients, the mean age of transplant recipients participating in the study was 53.7 ± 13.8 years. The most frequently used immunosuppressive drugs were tacrolimus (92.8%), mycophenolate mofetil (88.0%), and steroids (79.3%). Antihypertensives and antidiabetics were used in 88.0 and 27.4% of the patients, respectively. Approximately 20.9% of patients showed the presence of circulating donor-specific anti-HLA IgG antibodies at time of biopsy. Most of the samples (51.1%) were collected within the first 6 months after transplantation, 48.0% were protocol biopsies, followed by event-driven (43.6%), and follow-up biopsies (8.5%). Over time the proportion of biopsies classified into the categories Banff 4 (T-cell-mediated rejection [TCMR]) and Banff 1 (normal tissue) decreased whereas Banff 2 (antibody-mediated rejection [ABMR]) and Banff 5I (mild interstitial fibrosis and tubular atrophy) increased to 84.2 and 74.5%, respectively, after 4 years post transplantation. Patients with rejection showed worse kidney function than patients without rejection.

**Conclusion:** The clinical characteristics of subjects recruited indicate a patient cohort typical for routine renal transplantation all over Europe. A typical shift from T-cellular early rejections episodes to later antibody mediated allograft damage over time after renal transplantation further strengthens the usefulness of our cohort for the evaluation of novel biomarkers for allograft damage.

## Introduction

Despite a steady improvement of patient and organ survival after renal transplantation, allograft rejection continues to pose a risk of graft damage. In the first week and month after transplantation, T-cell-mediated rejection (TCMR) in particular is more common, while later antibody-mediated rejection (ABMR) accounts for the majority of immunological graft damages (1, 2).

Changes in kidney function, a decrease in urine output, or an increase in proteinuria may reflect transplant rejection during routine clinical patient care. Kidney biopsies are still current gold standard for diagnosing an allograft rejection, but as an invasive procedure it carries the risk of bleeding and other complications. The latter limits the routine use of serial biopsies, and the diagnosis of rejection is often made at an advanced stage of irreversible tissue injury. At many transplant units, protocol biopsies have been introduced to potentially detect the acute rejection already in a sub-clinical state (3). However, with serial biopsies, it is unlikely that all rejection episodes will be detected upon onset, not to speak of complications associated with such a costly approach (4).

Biomarkers in the urine could help to detect rejections early and non-invasively, whereby an appropriate sensitivity and specificity as well as a quick diagnosis are necessary for the clinical routine (5, 6).

Recently, we developed a novel, non-invasive method to detect the graft rejection *via* a characteristic constellation of the urine metabolites alanine, citrate, lactate, and urea investigated by NMR spectroscopy (7). In a first monocentric prospective observational (UMBRELLA) study which included 109 patients, the test performance reached an area under the curve (AUC_ROC_) value of 0.84 when combining metabolomic analysis and corresponding estimated glomerular filtration rate (eGFR) values at time of urine sampling (8).

The subsequently following PARASOL study presented here is an open, international, multicenter, prospective, observational study, in which the diagnostic accuracy for the urinary metabolite constellation initially assessed in the UMBRELLA study will be validated in an independent cohort resembling the routine kidney transplantation programs in six different European transplant centers. The study is based on a reasonable number of patients recruited with their respective urine samples and renal allograft biopsies, such as both protocol and event-driven biopsies.

## Materials and Methods

### Study Design

Urine samples were taken from adult (≥18 years) renal or combined renal and pancreas transplant patients prior to a kidney biopsy that was performed according to local center standards as protocol or event-driven biopsies. The target population is thus within the clinical routine and planned for renal allograft biopsies. The patient population consists of patients recruited at least 14 days after transplantation. To ensure broad real-world spectrum of biopsy results, all patients scheduled for a renal allograft biopsy were screened for eligibility.

**Figure 1:**
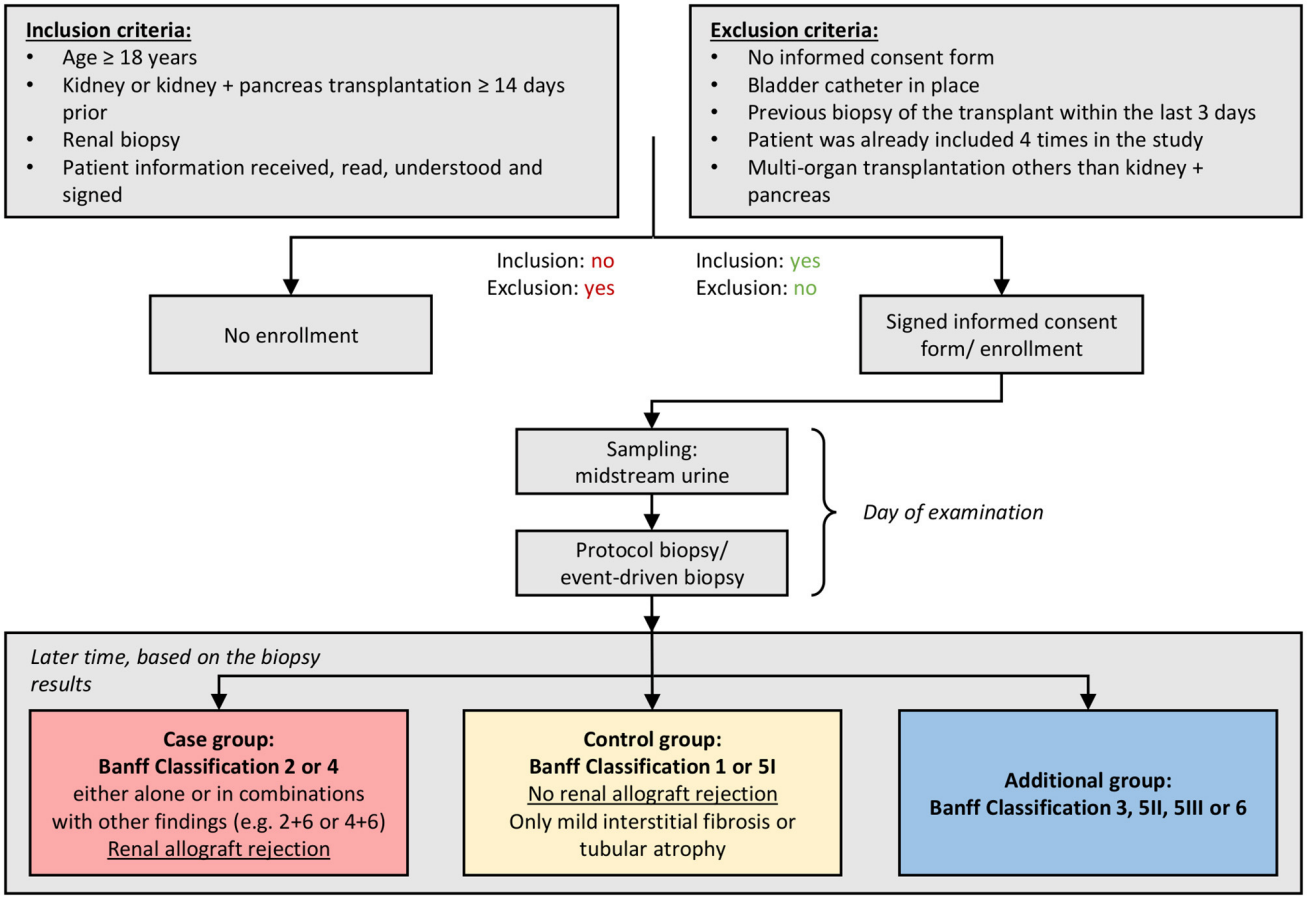
Study sequence. Patients who received a kidney or kidney and pancreas transplantation ≥14 days prior were selected according to the inclusion criteria of the PARASOL study. After informed consent discussion and signed informed consent form, the clinical data were recorded, the midstream urine sample was collected, and the kidney biopsy was taken within the clinical routine. According to biopsy results, patients were assigned either to a case group [Banff category 2 (antibody-mediated rejection [ABMR]) or 4 (T-cell-mediated rejection [TCMR]), either alone or in combination with other findings (other non-rejection changes)], a control group [(no rejection): Banff categories 1 (normal biopsy), 5I (mild interstitial fibrosis and atrophy (IFTA)], and an additional group [Banff categories 3 (suspicious for TCMR), 5II (moderate IFTA), 5III (severe IFTA), or 6 (other non-rejection changes)]. The study was then ended for the participant.

Between June 2017 and March 2020, 972 transplant recipients were included (1,230 urine samples and matched biopsies, respectively). According to biopsy results, patients were retrospectively assigned to a case group [Banff category 2 (ABMR) or 4 (TCMR), either alone or in combination with other findings (other non-rejection changes)], a control group [(no rejection): Banff categories 1 (normal biopsy), 5I (mild interstitial fibrosis and atrophy (IFTA)], and an additional group [Banff categories 3 (suspicious for TCMR), 5II (moderate IFTA), 5III (severe IFTA), or 6 (other non-rejection changes)]. Criteria for subject categorization—based on the rules of the Banff classification—are detailed in Figure 1.

Clinical routine shows that patients of the target population may be biopsied several times. In this context, PARASOL collected data on the day of the examination (cross-sectional data), but did not take any data over time (longitudinal data) into account. This resulted in the possibility that some patients were included in the study several times. These patients signed the informed consent forms again and new patient identification numbers were given (at a maximum of four times). The study sequence is similar to an initial recruitment (Table 1).

**Table 1:** Examination matrix.

**Assessments to be performed**	**On one day of the regular examinations, ≥14 days after kidney transplantation**	**Later time**	**Comment on timing**
Informed consent	X		Prior to any study procedure
Demography (a)	X		
Anamnesis (b)	X		Renal transplantation: ≥14 days before urine sample collection
			Last renal transplant biopsy: not within the last 3 days before sample collection.
			Last serum creatinine, Cystatin-C (optional), eGFR, CMV/ BKV Infection, Urinary tract infection: at day of (or max. 3 days prior to) urine sampling, during regular care (d)
Inclusion/ exclusion criteria	X		Criteria at day of urine sampling
Sampling of midstream urine	X		≥14 days after renal transplantation, before scheduled transplant biopsy, during regular care
Medication (c)	X		At time of urine sampling
Renal transplant biopsy	X		At same day of urine sampling, at a time point after urine sampling, during regular care
Biopsy result		X	
Final status		X	

*(a) Age at enrollment (date of signed ICF), ethnicity, and sex. (b) Date of (last) renal transplantation, date of last renal transplant biopsy, last serum creatinine, last cystatin-C (optional), last estimated glomerular filtration rate (eGFR), existence of donor-specific antibodies (DSA), active CMV infection (based on CMV virus detection via PCR at the time of taking the urine sample), active BKV infection (based on polyoma virus detection in the blood of a patient via PCR testing at the time of taking the urine sample and/or direct staining of polyoma virus in the corresponding renal biopsy), active urinary tract infection, diabetes mellitus, hypertension, and underlying renal disease. (c) Immunosuppressants [tacrolimus, cyclosporine, steroids, mycofenolate mofetil (MMF)/mycophenolic acid (MPA), other immunosuppressants], antibiotics, antihypertensives, and antidiabetics. (d) If status was determined >3 days before urine sampling, it is considered as “N/A”*.

To preclude an interaction between the biopsy procedure and the results of the NMR analysis samples were obtained before biopsies and, in case of two or more sequential biopsies, a sufficiently large interval between the biopsies was defined in the protocol to prevent possible blood residues in urine affecting the NMR analysis of the second sample. In addition, we defined an upper limit for re-recruitment of a patient. The goal was to include an adequate number of patients to get a representative reflection of the biological variance in the study population. Therefore, a maximum of four biopsies per patient could be included in the study. The patients, such as not willing to participate, with a bladder catheter in place, with a previous biopsy of the transplant within the last 3 days, already included four times in the study, and after multi-organ transplantation other than kidney and pancreas, are excluded from the study. Respective clinical data (e.g., date of transplantation, medication, donor-specific anti-HLA IgG antibodies (DSA), former rejections, and infections) were collected on the day of the examination and the urine sample was taken immediately before the planned biopsy. The report of the kidney histopathology analysis, as clinical reference standard, was added to the study documentation after becoming available. Definition of DSA was done individually according to local center standards. The threshold for the positivity was defined as 800–1,000 mean fluorescence intensity (MFI) depending on the local protocol and assay used. The centers used mixed and single antigen (SAB) class I and class II bead assays. No follow-up appointments were planned for the study participants.

The study was approved by the local Ethics Committees of all participating centers. The written informed consent was received from participants prior to study inclusion.

### Kidney Biopsies

The results of morphologic renal allograft evaluation were documented as the reference standard. The study included protocol biopsies (as per local center protocol), event-driven biopsies, as well as follow-up biopsies performed for monitoring of treatment responses. Specimens were evaluated in the context of center routine by experienced local renal pathologists following the rules of the 2018 Reference Guide to the Banff Classification of Renal Allograft Pathology (9). For analysis in relation to biomarker results, biopsies were grouped according to Banff diagnostic categories as follows: (i) case group (rejection): Banff category 2 (ABMR) or 4 (TCMR), either alone or in combination with other findings (other non-rejection changes) (ii) control group (no rejection): Banff categories 1 (normal biopsy), 5I (mild IFTA) (iii) additional group: [Banff categories 3 (suspicious for TCMR), 5II (moderate IFTA), 5III (severe IFTA), or 6 (other non-rejection changes)], respectively.

### NMR-Analysis

Spontaneous mid-stream urine samples were collected and measured by NMR spectroscopy at numares AG (Regensburg, Germany). For this, a volume of 600 μl of each urine sample was mixed with 150 μl of Axinon® urine additive solution in a centrifuge tube. The samples were centrifuged at 20,000 g for 10 min at 20°C, and 600 μl of the supernatant were transferred to 5 mm NMR tubes and kept at 2–6°C until measurement. All measurements were carried out on a Bruker Avance II + 600 MHz NMR-spectrometer as already described before (7). Using the Axinon® renalTX-SCORE® system, urinary metabolite quantification and test results were generated in a fully automated manner. The resulting score correlates with the probability of allograft rejection and ranges between 0 and 100 with low to high probability and is based on an NMR-based pattern encompassing the metabolites alanine, citrate, lactate, and urea.

### Statistical Analysis Strategy

Statistical analyses were planned in two ways: a descriptive analysis and a performance analysis. The former was to address the patient data and highlight demographic facts using clinical data derived from the corresponding case report form (CRF). It was planned that important variables, such as sex, age, ethnicity, and various disease factors of the patients would be investigated. Furthermore, all variables were considered to be used for the performance analysis.

It was clear that it may be necessary to exclude patient data from the performance analysis in case that technically no renalTX-SCORE output could be determined by NMR measurement. The performance analysis should use standard methods and assess conventional parameters for evaluating the quality of the diagnostic patterns developed. Particular attention to the area under the receiver operating characteristic (ROC) curve (AUC_ROC_), specificity, and sensitivity should be paid. In addition, certain subgroup performance analyses should be obtained in concerns of different cut-off values with regard to specificity and sensitivity.

### Sample Size Estimation

The number of cases was based on the observed values for the AUC_ROC_ values of the NMR-based patterns obtained in the UMBRELLA (8) study for the detection of acute renal allograft rejection (AUC_ROC_ = 0.75). In particular, the number of cases in the multicenter validation should be large enough to prove a difference between the AUC_ROC_ value of the developed NMR-based tests and a minimum AUC_ROC_ (null hypothesis H_0_) of 0.675 at the level of significance α = 5% with a power of 80%. The percentage of positive biopsy results (prevalence of acute Banff 4 renal allograft rejection) in the UMBRELLA collective was 27% (54/203 biopsies). The expected percentage of positive biopsy results in the PARASOL study was therefore estimated to be 30%. Accordingly, 151 rejections (cases) and 334 controls were needed. The indicated number of cases was calculated using the MedCalc v.12.7.7.0-64 bit, observing a two-tailed binomial test with a power of 80%. To compensate for the losses in recruiting, withdrawal of consent, insufficient urine quantities, losses, etc., a 15% safety margin was calculated so that recruiting should include 174 cases: renal allograft rejection Banff 2, 4, either alone or in combination with other findings, e.g., 2 + 6 or 4 + 6, and 384 controls: Banff classification 1 and 5I.

The estimated number of cases was exceeded with 237 cases instead of estimated 174 cases and 541 controls instead of the planned 384.

### Primary Endpoint

The primary endpoint of the study is the respective AUC_ROC_ value of the ROC curve for the NMR-based pattern. Acute renal allograft rejection is defined according to the Banff classification and categorized 2 (ABMR), 4 (TCMR), either alone or in combinations with other findings, e.g., 2 + 6 or 4 + 6. The AUC_ROC_ values and their 95% *CI*s are determined as a measure of diagnostic accuracy. It should be tested whether the diagnostic accuracy is to be proven with an AUC_ROC_ value of at least 0.675 under the assumption that the observed AUC_ROC_ value itself is 0.75.

### Secondary Endpoint

As a secondary endpoint, the recruited collective will be analyzed with respect to the determination of cut-off values associated with a sensitivity and specificity of 90% and the respective specificity at 90% sensitivity and sensitivity at 90% specificity including the 95% *CI* for the exact definition of the NMR-based pattern to detect renal allograft rejection. In addition, for the cut-off values determined, the sensitivities and specificities in patients with Banff classification 3, 5II, 5III, and 6 will be determined in a subgroup analysis if enough samples are available. Furthermore, the demographic and clinical parameters will be used to examine whether individual parameters or a group of these parameters are suitable for reliably predicting the occurrence of renal allograft rejection in this large, non-stratified patient group.

## Results

In the context of PARASOL (multicenter observational prospective trial including six European transplant centers), a total of 1,230 urine samples for metabolite evaluation and corresponding histopathology results (972 patients; 1.3 samples per patient) were collected (recruitment period between June 2017 and March 2020).

Table 2 provides sample and patient characteristics for the overall cohort and individual centers. Among 1,230 biopsy specimens, 237 samples (19.3%) were assigned to the case group, 541 to the control group (44.0%), and 452 (36.7%) samples were assigned to the additional group. Overall, 810 samples (65.9%) were obtained from male patients and 420 (34.1%) from female patients. The mean recipient age at the time of study inclusion was 53.7 ± 13.8 years (range 19–84 years) and most of the patients were Caucasian (96.3%).

**Table 2:** Urine sample characteristics collected by the six different study centers.

		**Total**	**Barcelona Bel**.	**Barcelona FP**.	**Grenoble**	**Praha**	**Vienna**	**Regensburg**
Number of patients	Total	972	21	49	348	150	219	185
	With one urine sample	790/972 (81.3%)	21/21 (100.0%)	47/49 (95.9%)	256/348 (73.6%)	138/150 (92.0%)	209/219 (95.4%)	119/185 (64.3%)
	With multiple urine samples	182/972 (18.7%)	0/21 (0.0%)	2/49 (4.1%)	92/348 (26.4%)	12/150 (8.0%)	10/219 (5.6%)	66/185 (35.7%)
Number of urine samples	Total	1,230	21	51	479	164	229	286
Urine samples per patient	Mean	1.3	1.0	1.0	1.4	1.1	1.0	1.5
Status	Case	237/1,230 (19.3%)	1/21 (4.8%)	4/51 (7.8%)	73/479 (15.2%)	60/164 (36.6%)	41/229 (17.9%)	58/286 (20.3%)
	Control	541/1,230 (44.0%)	15/21 (71.4%)	23/51 (45.1%)	260/479 (54.3%)	34/164 (20.7%)	96/229 (41.9%)	113/286 (39.5%)
	Additional	452/1,230 (36.7%)	5/21 (23.8%)	24/51 (47.1%)	146/479 (30.5%)	70/164 (42.7%)	92/229 (40.2%)	115/286 (40.2%)
Age	Range	19–84	37–78	26–75	21–84	21–82	20–79	19–84
	Mean ± SD	53.7 ± 13.8	58.7 ± 11.1	55.0 ± 13.3	54.2 ± 14.9	53.2 ± 13.2	54.3 ± 12.8	52.3 ± 13.2
Sex	Male	810/1,230 (65.9%)	12/21 (57.1%)	26/51 (51.0%)	311/479 (64.9%)	116/164 (70.7%)	151/229 (65.9%)	194/286 (67.8%)
	Female	420/1,230 (34.1%)	9/21 (42.9%)	25/51 (49.0%)	168/479 (35.1%)	48/164 (29.3%)	78/229 (34.1%)	92/286 (32.2%)
Ethnicity	African	24/1,230 (2.0%)	0/21 (0.0%)	1/51 (2.0%)	21/479 (4.4%)	0/164 (0.0%)	2/229 (0.9%)	0/286 (0.0%)
	Asian	8/1,230 (0.7%)	0/21 (0.0%)	0/51 (0.0%)	6/479 (1.3%)	1/164 (0.6%)	1/229 (0.4%)	0/286 (0.0%)
	Caucasian	1,184/1,230 (96.3%)	21/21 (100.0%)	45/51 (88.2%)	450/479 (93.9%)	162/164 (98.8%)	226/229 (98.7%)	280/286 (97.9%)
	Other	13/1,230 (1.1%)	0/21 (0.0%)	5/51 (9.8%)	2/479 (0.4%)	0/164 (0.0%)	0/229 (0.0%)	6/286 (2.1%)
	Not specified	1/1,230 (0.1%)	0/21 (0.0%)	0/51 (0.0%)	0/479 (0.0%)	1/164 (0.6%)	0/229 (0.0%)	0/286 (0.0%)
Biopsy reason	Event-driven	536/1,230 (43.6%)	8/21 (38.1%)	15/51 (29.4%)	164/479 (34.2%)	114/164 (69.5%)	97/229 (42.4%)	138/286 (48.3%)
	Follow-up	104/1,230 (8.5%)	0/21 (0.0%)	1/51 (2.0%)	67/479 (14.0%)	0/164 (0.0%)	4/229 (1.7%)	32/286 (11.2%)
	Protocol	590/1,230 (48.0%)	13/21 (61.9%)	35/51 (68.6%)	248/479 (51.8%)	50/164 (30.5%)	128/229 (55.9%)	116/286 (40.6%)
Time after TX	≤ 6 months	628/1,230 (51.1%)	8/21 (38.1%)	20/51 (39.2%)	238/479 (49.7%)	92/164 (56.1%)	113/229 (49.3%)	157/286 (54.9%)
	[6,12] months	132/1,230 (10.7%)	4/21 (19.0%)	15/51 (29.4%)	60/479 (12.5%)	16/164 (9.8%)	22/229 (9.6%)	15/286 (5.2%)
	[1,4] years	222/1,230 (18.0%)	6/21 (28.6%)	11/51 (21.6%)	86/479 (18.0%)	29/164 (17.7%)	59/229 (25.8%)	31/286 (10.8%)
	>4 years	248/1,230 (20.2%)	3/21 (14.3%)	5/51 (9.8%)	95/479 (19.8%)	27/164 (16.5%)	35/229 (15.3%)	83/286 (29.0%)
Anamnesis	Infections	175/1,230 (14.2%)	1/21 (4.8%)	5/51 (9.8%)	101/479 (21.1%)	16/164 (9.8%)	39/229 (17.0%)	13/286 (4.5%)
	Diabetes	364/1,230 (29.6%)	8/21 (38.1%)	9/51 (17.6%)	123/479 (25.7%)	56/164 (34.1%)	62/229 (27.1%)	106/286 (37.1%)
	Hypertension	1,092/1,230 (88.8%)	17/21 (81.0%)	48/51 (94.1%)	389/479 (81.2%)	149/164 (90.9%)	211/229 (92.1%)	278/286 (97.2%)
Medication	Tacrolimus	1,141/1,230 (92.8%)	20/21 (95.2%)	50/51 (98.0%)	429/479 (89.6%)	152/164 (92.7%)	216/229 (94.3%)	274/286 (95.8%)
	Cyclosporine	31/1,230 (2.5%)	0/21 (0.0%)	0/51 (0.0%)	8/479 (1.7%)	5/164 (3.0%)	11/229 (4.8%)	7/286 (2.4%)
	Steroids	975/1,230 (79.3%)	18/21 (85.7%)	51/51 (100.0%)	317/479 (66.2%)	156/164 (95.1%)	224/229 (97.8%)	209/286 (73.1%)
	MMF/MPA	1,082/1,230 (88.0%)	21/21 (100.0%)	42/51 (82.4%)	389/479 (81.2%)	138/164 (84.1%)	217/229 (94.8%)	275/286 (96.2%)
	Antibiotics	306/1,230 (24.9%)	4/21 (19.0%)	2/51 (3.9%)	107/479 (22.3%)	31/164 (18.9%)	83/229 (36.2%)	79/286 (27.6%)
	Antihyper-tensives	1,083/1,230 (88.0%)	17/21 (81.0%)	48/51 (94.1%)	386/479 (80.6%)	147/164 (89.6%)	209/229 (91.3%)	276/286 (96.5%)
	Antidiabetics	337/1,230 (27.4%)	8/21 (38.1%)	12/51 (23.5%)	123/479 (25.7%)	52/164 (31.7%)	62/229 (27.1%)	80/286 (28.0%)

*Overall and for each center respectively, the distribution for different subsets is shown. The numbers and relative frequencies in the categories status, age, sex, ethnicity, biopsy type, time after renal transplantation (TX), anamnesis, and medication refer to the number of urine samples. Infections include active CMV (based on CMV virus detection via PCR at the time of taking the urine sample), active BKV (based on polyoma virus detection in the blood of a patient via PCR testing at the time of taking the urine sample and/or direct staining of polyoma virus in the corresponding renal biopsy) and/or active urinary tract infections. Case group: Banff category 2 (ABMR [antibody-mediated rejection]) or 4 (T-cell-mediated rejection [TCMR]), either alone or in combination with other findings (other non-rejection changes); control group [(no rejection): Banff categories 1 (normal biopsy), 5I (mild interstitial fibrosis and atrophy (IFTA)]; additional group [Banff categories 3 (suspicious for TCMR), 5II (moderate IFTA), 5III (severe IFTA), or 6 (other non-rejection changes)]. For further clarification: by study design, at day of urine sample collection a biopsy was performed*.

Tacrolimus (92.8%), mycophenolate mofetil (88.0%), and steroids (79.3%) were the most frequently used immunosuppressive drugs. Antihypertensives and antidiabetics were used in 88.0 and 27.4% of the patients, respectively. About 88.8% of patients suffered from hypertension, 29.6% were diabetics, and 14.2% had any infection (BKV, CMV, or urinary tract infection) at the time of inclusion into the study.

Of the 1,230 urine samples, 628 (51.1%) were collected within the first 6 months after transplantation, 132 (10.7%) between 6 and 12 months, 222 (18.0%) between 1 and 4 years, and 248 (20.2%) more than 4 years after transplantation. Most of the samples [590 (48.0%)] were in the context of protocol biopsies, followed by event-driven biopsies [536 (43.6%)], and follow-up biopsies [104 (8.5%)].

**Figure 2:**
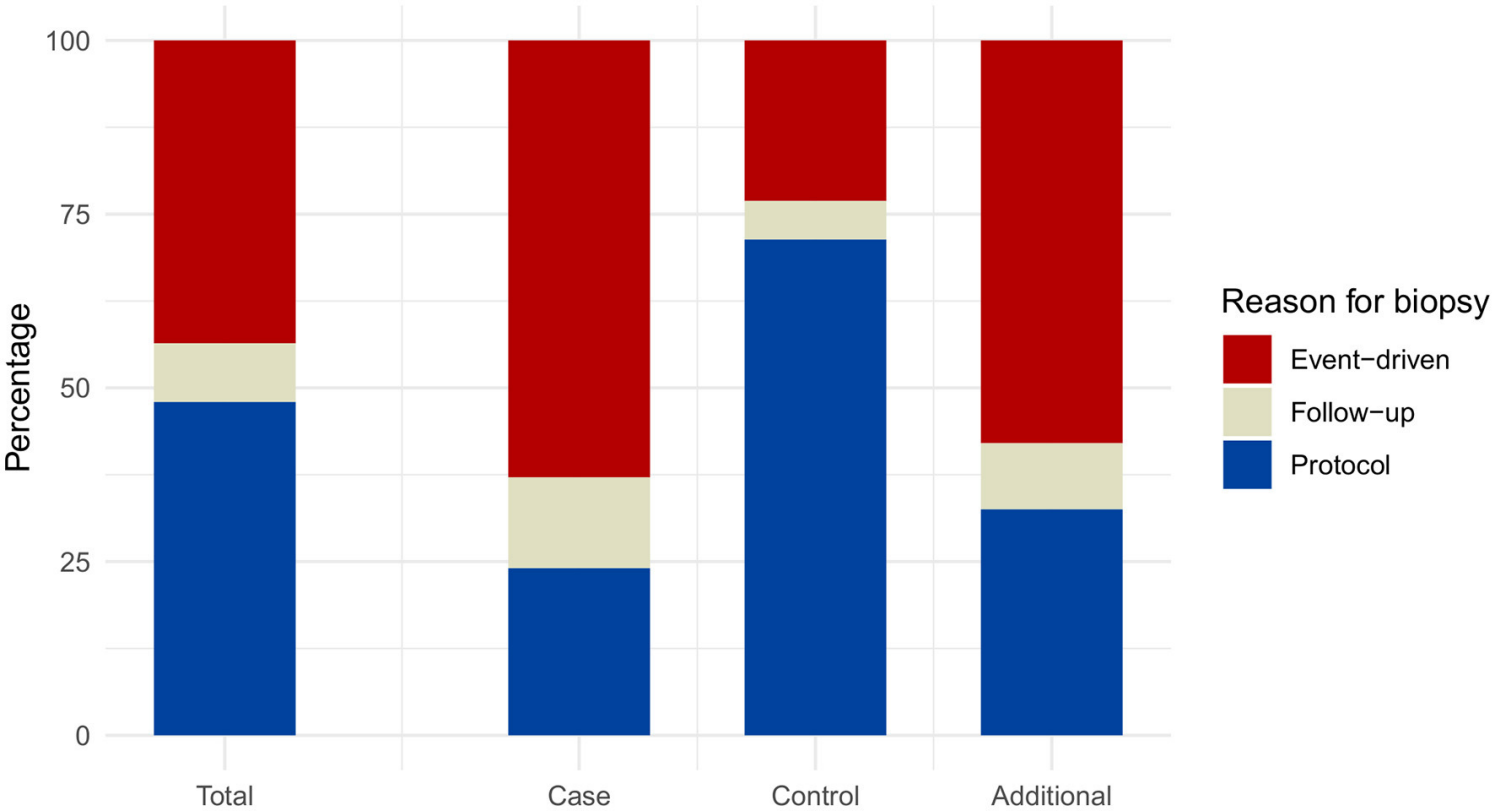
Distribution of the reasons for biopsy. Relative frequencies are displayed for the total number of samples (irrespective of the group assignment), as well as for each of the status groups. Case group: Banff category 2 (ABMR) or 4 (TCMR), either alone or in combination with other non-rejection changes; Control group: Banff categories 1 (normal biopsy), 5I (mild IFTA); Additional group: Banff categories 3 (suspicious for TCMR), 5II (moderate IFTA), 5III (severe IFTA), or 6 (other non-rejection changes).

The distribution of biopsy types for case, control, and additional group differed in the groups. In the case group, 62.9% event-driven biopsies were noted, 24.1% protocol biopsies, and 13.1% follow-up biopsies. In the control group, 71.3% protocol biopsies, 23.1% event-driven biopsies, and 5.5% follow-up biopsies were seen. The additional group consisted of 58.0% event-driven biopsies, 32.5% protocol biopsies, and 9.5% follow-up biopsies (Figure 2 and Supplementary Table S1). Additionally Supplementary Figure S1 and Supplementary Table S2 show the distribution of the case, control, and additional groups within each of the three biopsy reasons: event-driven, follow-up, and protocol.

### Distribution of Banff Categories Within Case (Rejection), Control, and Additional Group

**Figure 3:**
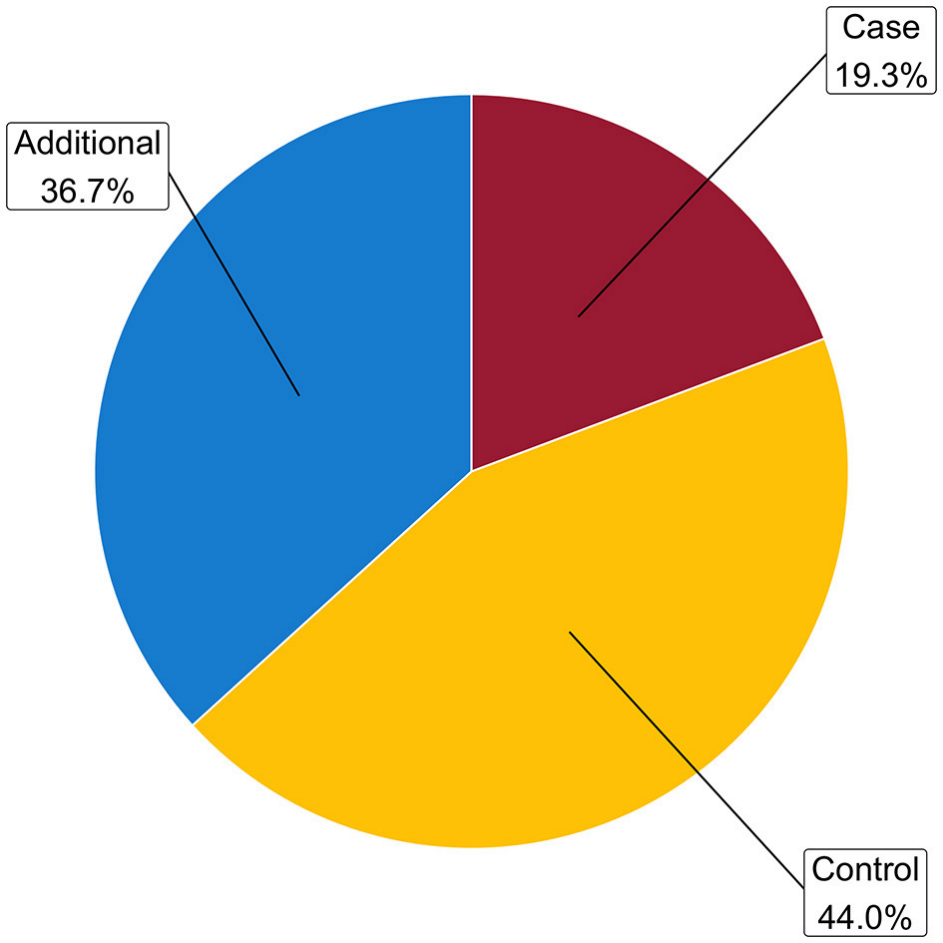
Distribution of the status groups. Relative frequencies are derived from the total number of samples. Case group: Banff category 2 (ABMR) or 4 (TCMR), either alone or in combination with other findings (other non-rejection changes); control group [(no rejection): Banff categories 1 (normal biopsy), 5I (mild IFTA); additional group (Banff categories 3 (suspicious for TCMR), 5II (moderate IFTA), 5III (severe IFTA), or 6 (other non-rejection changes)].

Most of the biopsies (44.0%) were assigned to the control group, corresponding histopathology showed classification into the Banff categories 1 (normal biopsy) (28.0% out of all biopsies) and Banff 5I (mild IFTA) (16.0% out of all biopsies) for the largest proportion of findings, followed by other combinations (15.2%) and allograft fibrosis classified as Banff grades 5 II (moderate IFTA) and III (severe IFTA) (10.0%) both belonging to the additional group (Figure 3 and Supplementary Table S3).

**Figure 4:**
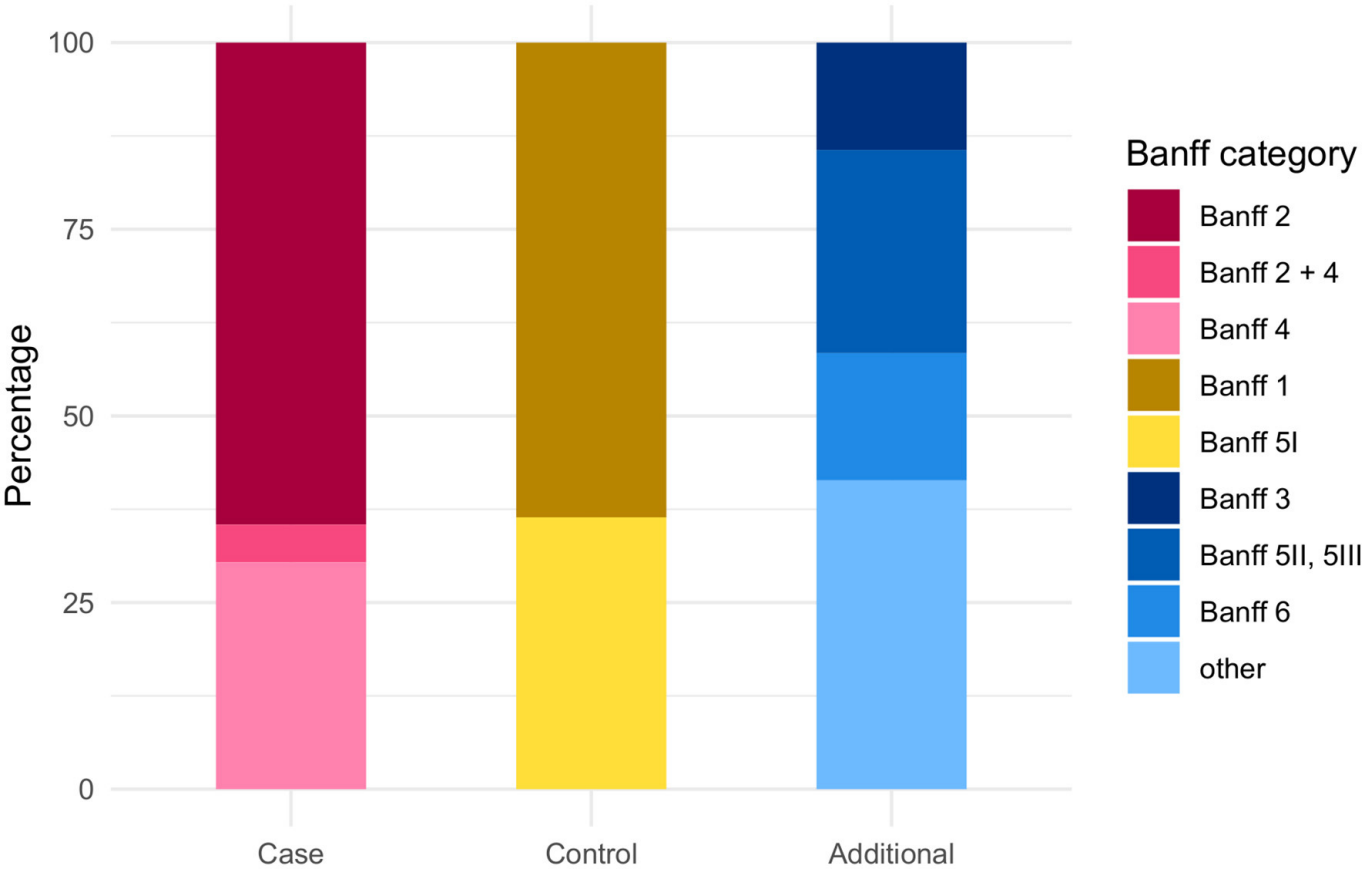
Distribution of Banff categories among the case, control, and additional group. Case group: Banff category 2 (ABMR) or 4 (TCMR), either alone or in combination with other non-rejection changes; Control group: Banff categories 1 (normal biopsy), 5I (mild IFTA); Additional group: Banff categories 3 (suspicious for TCMR), 5II (moderate IFTA), 5III (severe IFTA), or 6 (other non-rejection changes).

Within the group of rejection, ABMR (Banff 2) represented the largest proportion (64.6%), followed by TCMR (Banff 4: 30.4%). Combined ABMR and TCMR was infrequent (5.1%) (Figure 4 and Supplementary Table S3). The control group showed further a higher proportion of Banff 1 (63.6%) than of Banff 5I (36.4%) graded biopsies (Figure 4 and Supplementary Table S3). In the additional group, the highest proportion of pathology findings belongs to other combinations (41.4%), Banff 5II and III (27.2%), followed by Banff 6 (17.0%) and Banff 3 (14.4%) (Figure 4 and Supplementary Table S3).

### Type of Renal Allograft Rejections Dependent From Time After Transplantation

**Figure 5:**
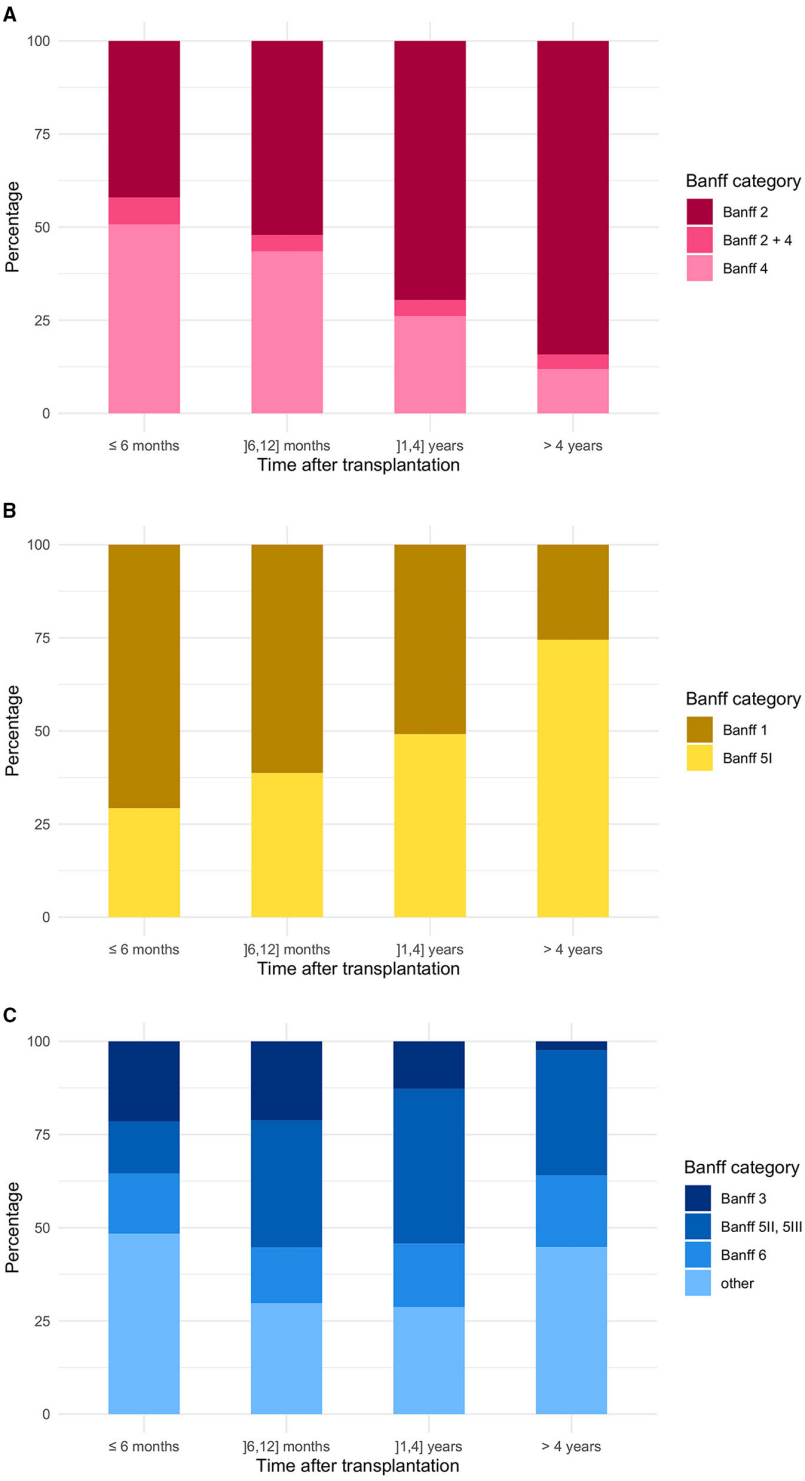
Banff category distribution in concerns of time after renal transplantation **(A)** in the case group, **(B)** in the control group, and **(C)** in the additional group. Case group: Banff category 2 (ABMR) or 4 (TCMR), either alone or in combination with other non-rejection changes; Control group: Banff categories 1 (normal biopsy), 5I (mild IFTA); Additional group: Banff categories 3 (suspicious for TCMR), 5II (moderate IFTA), 5III (severe IFTA), or 6 (other non-rejection changes).

Within the case group, ABMR classified as Banff 2 category (either isolated or in combination with other lesions) represented a large group of rejections with 42.0% already within the first 6 months post transplantation. With increasing time post-transplantation, the proportion of antibody mediated rejection increased up to 84.2% (after 4 years). TCMR (Banff 4 and Banff 4 + x) decreased from 50.7 to 11.8% over time. The combination of both stayed stable with 7.3% and 4.0% (Figure 5A and Supplementary Table S4).

In the control group within the first 6 months, most biopsy samples were assigned to Banff 1 (70.8%), whereas after 4 years, almost 75% of biopsies of the control group were classified as Banff 5I (Figure 5B and Supplementary Table S4).

In the additional group, the proportion of borderline rejections (Banff 3) decreased from 21.5 to 2.4% over time, whereas the amount of graft fibrosis represented by Banff 5II and III findings increased to 33.6% (Figure 5C and Supplementary Table S4).

### Detection of Donor-Specific Antibodies (DSA) Dependent on Sample Classification and Banff Categories

For 257 of the 1,230 samples, positive corresponding DSA results were documented (20.9%) (Table 3). Within the case group for 43.5% of the samples (103/237), DSAs were noted. 84 from these samples (81.6%) showed ABMR (Banff 2). Simultaneous presence of DSA in blood was documented for only 80/541 samples (14.8%) from the control group patients. Therein, in the subgroup associated with Banff 1 findings in the biopsy 46/80 (57.5%) samples corresponded to patients with DSA, in the subgroup with Banff 5I lesion, the rate was 34/80 (42.5%). In the additional group, 74/452 (16.4%) samples were associated with DSA.

**Table 3:** Existence of donor-specific anti-HLA IgG antibodies (DSA) in the status groups and each of the Banff categories.

		**Total**	**DSA negative**	**DSA positive**	**DSA not specified**
Number of samples		1,230	802	257	171
Status: case	Total	237	106/237 (44.7%)	103/237 (43.5%)	28/237 (11.8%)
	Banff 2	153/237 (64.6%)	45/153 (29.4%)	84/153 (54.9%)	24/153 (15.7%)
	Banff 2 + 4	12/237 (5.1%)	5/12 (41.7%)	7/12 (58.3%)	0/12 (0.0%)
	Banff 4	72/237 (30.4%)	56/72 (77.8%)	12/72 (16.7%)	4/72 (5.6%)
Status: control	Total	541	395/541 (73.0%)	80/541 (14.8%)	66/541 (12.2%)
	Banff 1	344/541 (63.6%)	258/344 (75.0%)	46/344 (13.4%)	40/344 (11.6%)
	Banff 5I	197/541 (36.4%)	137/197 (69.5%)	34/197 (17.3%)	26/197 (13.2%)
Status: additional	Total	452	301/452 (66.6%)	74/452 (16.4%)	77/452 (17.0%)
	Banff 3	65/452 (14.4%)	41/65 (63.1%)	15/65 (23.1%)	9/65 (13.8%)
	Banff 5II, 5III	123/452 (27.2%)	68/123 (55.3%)	27/123 (22.0%)	28/123 (22.8%)
	Banff 6	77/452 (17.0%)	58/77 (75.3%)	4/77 (5.2%)	15/77 (19.5%)
	Other	187/452 (41.4%)	134/187 (71.7%)	28/187 (15.0%)	25/187 (13.4%)

*Relative frequencies are derived from the respective total number of samples in the three status groups. The order of the Banff categories is based on the assignment to case, control, and additional. Case group: Banff category 2 (ABMR) or 4 (TCMR), either alone or in combination with other non-rejection changes; Control group: Banff categories 1 (normal biopsy), 5I (mild IFTA); Additional group: Banff categories 3 (suspicious for TCMR), 5II (moderate IFTA), 5III (severe IFTA), or 6 (other non-rejection changes)*.

### Distribution of Chronic Kidney Disease (CKD) Stages Among Status, Time After Renal Transplantation, and Recipient Age

Kidney function was seen to be dependent on the status (case/control/additional), time after transplantation, and the recipient age.

Regarding the severity of CKD of the study population, patients with allograft rejections and from the additional group showed significantly worse kidney function than controls (samples 318 corresponding with CKD stage 4 or 5 32.9% in cases vs. 11.4% in controls vs. 32.6% in the additional group; Table 4). Whereas control group samples were associated with good allograft function up to 4 years, case group samples and additional group samples corresponded with worse kidney function (Table 4).

**Table 4:** Distribution of chronic kidney disease (CKD) stages among status groups and time after renal transplantation (TX).

		**Total**	**CKD Stage 1**	**CKD Stage 2**	**CKD Stage 3A**	**CKD Stage 3B**	**CKD Stage 4**	**CKD Stage 5**	**CKD Stage not specified**
Number of samples		1,230	34	193	253	288	235	52	175
Status: case	Total	237	2/237 (0.8%)	31/237 (13.1%)	36/237 (15.2%)	62/237 (26.2%)	58/237 (24.5%)	20/237 (8.4%)	28/237 (11.8%)
	≤ 6 months	69/237 (29.1%)	1/69 (1.4%)	16/69 (23.2%)	9/69 (13.0%)	19/69 (27.5%)	10/69 (14.5%)	7/69 (10.1%)	7/69 (10.1%)
	[6,12] months	23/237 (9.7%)	1/23 (4.3%)	3/23 (13.0%)	2/23 (8.7%)	7/23 (30.4%)	6/23 (26.1%)	2/23 (8.7%)	2/23 (8.7%)
	[1,4] years	69/237 (29.1%)	0/69 (0.0%)	9/69 (13.0%)	11/69 (15.9%)	19/69 (27.5%)	20/69 (29.0%)	2/69 (2.9%)	8/69 (11.6%)
	>4 years	76/237 (32.1%)	0/76 (0.0%)	3/76 (3.9%)	14/76 (18.4%)	17/76 (22.4%)	22/76 (28.9%)	9/76 (11.8%)	11/76 (14.5%)
Status: control	Total	541	22/541 (4.1%)	115/541 (21.3%)	137/541 (25.3%)	116/541 (21.4%)	52/541 (9.6%)	10/541 (1.8%)	89/541 (16.5%)
	≤ 6 months	373/541 (68.9%)	15/373 (4.0%)	88/373 (23.6%)	110/373 (29.5%)	83/373 (22.3%)	28/373 (7.5%)	7/373 (1.9%)	42/373 (11.3%)
	[6,12] months	62/541 (11.5%)	4/62 (6.5%)	12/62 (19.4%)	12/62 (19.4%)	12/62 (19.4%)	7/62 (11.3%)	1/62 (1.6%)	14/62 (22.6%)
	[1,4] years	59/541 (10.9%)	3/59 (5.1%)	10/59 (16.9%)	10/59 (16.9%)	10/59 (16.9%)	6/59 (10.2%)	1/59 (1.7%)	19/59 (32.2%)
	>4 years	47/541 (8.9%)	0/47 (0.0%)	5/47 (10.6%)	5/47 (10.6%)	11/47 (23.4%)	11/47 (23.4%)	1/47 (2.1%)	14/47 (29.8%)
Status: additional	Total	452	10/452 (2.2%)	47/452 (10.4%)	80/452 (17.7%)	110/452 (24.3%)	125/452 (27.7%)	22/452 (4.9%)	58/452 (12.8%)
	≤ 6 months	186/452 (41.2%)	7/186 (3.8%)	26/186 (14.0%)	35/186 (18.8%)	54/186 (29.0%)	46/186 (24.7%)	9/186 (4.8%)	9/186 (4.8%)
	[6,12] months	47/452 (10.4%)	3/47 (6.4%)	5/47 (10.6%)	13/47 (27.7%)	7/47 (14.9%)	9/47 (19.1%)	4/47 (8.5%)	6/47 (12.8%)
	[1,4] years	94/452 (20.8%)	0/94 (0.0%)	7/94 (7.4%)	17/94 (18.1%)	27/94 (28.7%)	25/94 (26.6%)	3/94 (3.2%)	15/94 (16.0%)
	>4 years	125/452 (27.7%)	0/125 (0.0%)	9/125 (7.2%)	15/125 (12.0%)	22/125 (17.6%)	45/125 (36.0%)	6/125 (4.8%)	28/125 (22.4%)

*The relative frequencies are derived from the respective total number of samples in each status group. However, the relative frequencies for each time after transplantation category in the respective status groups are computed using the total number of samples in the respective time after transplantation category. Samples that did not have an eGFR value assigned are included in the “CKD not specified” group. Case group: Banff category 2 (ABMR) or 4 (TCMR), either alone or in combination with other non-rejection changes; Control group: Banff categories 1 (normal biopsy), 5I (mild IFTA); Additional group: Banff categories 3 (suspicious for TCMR), 5II (moderate IFTA), 5III (severe IFTA), or 6 (other non-rejection changes)*.

With regards to the allograft recipient age, it could be seen that controls showed a better renal function within all age groups compared with cases and samples from the additional group (Table 5).

**Table 5:** Distribution of CKD stages in different age groups shown for case, control and additional group.

		**Total**	**19–29 years**	**30–39 years**	**40–49 years**	**50–59 years**	**60–69 years**	**70–79 years**	**80–84 years**
Number of samples		1,230	73	141	243	306	297	158	12
Status: case	Total	237	27/237 (11.4%)	27/237 (11.4%)	66/237 (27.8%)	48/237 (20.3%)	48/237 (20.3%)	18/237 (7.6%)	3/237 (1.3%)
	CKD Stage 1	2/237 (0.8%)	0/2 (0.0%)	1/2 (50.0%)	1/2 (50.0%)	0/2 (0.0%)	0/2 (0.0%)	0/2 (0.0%)	0/2 (0.0%)
	CKD Stage 2	31/237 (13.1%)	9/31 (29.0%)	3/31 (9.7%)	12/31 (38.7%)	4/31 (12.9%)	2/31 (6.5%)	1/31 (3.2%)	0/31 (0.0%)
	CKD Stage 3A	36/237 (15.2%)	6/36 (16.7%)	6/36 (16.7%)	11/36 (30.6%)	6/36 (16.7%)	6/36 (16.7%)	1/36 (2.8%)	0/36 (0.0%)
	CKD Stage 3B	62/237 (26.2%)	7/62 (11.3%)	7/62 (11.3%)	14/62 (22.6%)	12/62 (19.4%)	16/62 (25.8%)	5/62 (8.1%)	1/62 (1.6%)
	CKD Stage 4	58/237 (24.5%)	5/58 (8.6%)	8/58 (13.8%)	12/58 (20.7%)	12/58 (20.7%)	12/58 (20.7%)	7/58 (12.1%)	2/58 (3.4%)
	CKD Stage 5	20/237 (8.4%)	0/20 (0.0%)	0/20 (0.0%)	8/20 (40.0%)	6/20 (30.0%)	5/20 (25.0%)	1/20 (5.0%)	0/20 (0.0%)
	CKD Stage not specified	28/237 (11.8%)	0/28 (0.0%)	2/28 (7.1%)	8/28 (28.6%)	8/28 (28.6%)	7/28 (25.0%)	3/28 (10.7%)	0/28 (0.0%)
Status: control	Total	541	22/541 (4.1%)	64/541 (11.8%)	96/541 (17.7%)	140/541 (25.9%)	139/541 (25.7%)	75/541 (13.9%)	5/541 (0.9%)
	CKD Stage 1	22/541 (4.1%)	3/22 (13.6%)	7/22 (31.8%)	2/22 (9.1%)	5/22 (22.7%)	5/22 (22.7%)	0/22 (0.0%)	0/22 (0.0%)
	CKD Stage 2	115/541 (21.3%)	6/115 (5.2%)	22/115 (19.1%)	25/115 (21.7%)	27/115 (23.5%)	23/115 (20.0%)	10/115 (8.7%)	2/115 (1.7%)
	CKD Stage 3A	137/541 (25.3%)	4/137 (2.9%)	16/137 (11.7%)	22/137 (16.1%)	46/137 (33.6%)	33/137 (24.1%)	15/137 (10.9%)	1/137 (0.7%)
	CKD Stage 3B	116/541 (21.4%)	5/116 (4.3%)	7/116 (6.0%)	17/116 (14.7%)	25/116 (21.6%)	37/116 (31.9%)	24/116 (20.7%)	1/116 (0.9%)
	CKD Stage 4	52/541 (9.6%)	2/52 (3.8%)	4/52 (7.7%)	10/52 (19.2%)	10/52 (19.2%)	15/52 (28.8%)	10/52 (19.2%)	1/52 (1.9%)
	CKD Stage 5	10/541 (1.8%)	0/10 (0.0%)	0/10 (0.0%)	1/10 (10.0%)	2/10 (20.0%)	4/10 (40.0%)	3/10 (30.0%)	0/10 (0.0%)
	CKD Stage not specified	89/541 (16.5%)	2/89 (2.2%)	8/89 (9.0%)	19/89 (21.3%)	25/89 (28.1%)	22/89 (24.7%)	13/89 (14.6%)	0/89 (0.0%)
Status: additional	Total	452	24/452 (5.3%)	50/452 (11.1%)	81/452 (17.9%)	118/452 (26.1%)	110/452 (24.3%)	65/452 (14.4%)	4/452 (0.9%)
	CKD Stage 1	10/452 (2.2%)	3/10 (30.0%)	1/10 (10.0%)	3/10 (30.0%)	2/10 (20.0%)	1/10 (10.0%)	0/10 (0.0%)	0/10 (0.0%)
	CKD Stage 2	47/452 (10.4%)	4/47 (8.5%)	11/47 (23.4%)	6/47 (12.8%)	16/47 (34.0%)	6/47 (12.8%)	4/47 (8.5%)	0/47 (0.0%)
	CKD Stage 3A	80/452 (17.7%)	4/80 (5.0%)	10/80 (12.5%)	22/80 (27.5%)	20/80 (25.0%)	13/80 (16.2%)	11/80 (13.8%)	0/80 (0.0%)
	CKD Stage 3B	110/452 (24.3%)	4/110 (3.6%)	8/110 (7.3%)	19/110 (17.3%)	30/110 (27.3%)	32/110 (29.1%)	17/110 (15.5%)	0/110 (0.0%)
	CKD Stage 4	125/452 (27.7%)	3/125 (2.4%)	10/125 (8.0%)	20/125 (16.0%)	32/125 (25.6%)	37/125 (29.6%)	20/125 (16.0%)	3/125 (2.4%)
	CKD Stage 5	22/452 (4.9%)	0/22 (0.0%)	4/22 (18.2%)	3/22 (13.6%)	4/22 (18.2%)	6/22 (27.3%)	4/22 (18.2%)	1/22 (4.5%)
	CKD Stage not specified	58/452 (12.8%)	6/58 (10.3%)	6/58 (10.3%)	8/58 (13.8%)	14/58 (24.1%)	15/58 (25.9%)	9/58 (15.5%)	0/58 (0.0%)

*Within each of the status groups, the bold numbers and relative frequencies refer to the total number of samples in that group. However, the relative frequencies for each age group in the respective CKD stages are calculated using the total number of samples in the respective age group within the status group. Samples that did not have an eGFR value assigned are included in the CKD “not specified” group. Case group: Banff category 2 (ABMR) or 4 (TCMR), either alone or in combination with other non-rejection changes; Control group: Banff categories 1 (normal biopsy), 5I (mild IFTA); Additional group: Banff categories 3 (suspicious for TCMR), 5II (moderate IFTA), 5III (severe IFTA), or 6 (other non-rejection changes)*.

## Discussion

For patients with end stage renal disease, kidney transplantation is the renal replacement therapy of choice as it significantly improves the life quality and life expectancy (10). However, by far not all suitable patients can be offered a donor kidney as both post-mortal and living kidney donation are limited. With the observed increase in patients in the majority of countries worldwide suffering from renal disease, the gap between donor kidneys available and renal transplantation needed is more and more widening (11–13). One of the reasons of this observation is that renal allograft survival is limited and often shorter as the survival of recipient. In consequence, increasing numbers of patients with end stage renal disease need more than only one kidney transplantation and in countries with a very high rate of post-mortal organ donors, such as Spain, loss of a previous allograft is nowadays one of the main causes for listing patients for renal transplantation (14–23)^1^.

Therefore, one of the major goals in transplant medicine is to prolong the time of allograft survival, especially as more and more allografts with extended donor criteria have to be used to overcome organ shortage (2, 24–28). Despite major improvements in transplant patient care and therein immunosuppressive treatment, acute and chronic rejection processes often irreversibly damaging renal allograft tissues are still a major risk for premature allograft loss (1, 2, 29). Strategies to completely prevent allograft rejection are still missing, and diagnostic procedures for early detection of rejection are still suboptimal.

Until now, a histopathological evaluation of a renal allograft biopsy is the gold standard to detect the cause of an allograft malfunction after extra renal problems, such as perfusion deficits or urinary obstruction could be ruled out (1, 3, 4). Molecular analyses of renal tissue samples become more and more important and look very promising to become routine in the future, however, so far in many countries, this technique is not available due to its costs not routinely covered by medical insurance companies.

Several biomarkers have been previously described mainly in blood samples to detect allograft damage without performing renal biopsies, and costs and insufficient proof of usefulness under routine conditions are among the reasons for only limited use in daily clinical practice so far (5, 6, 30–35).

A characteristic urinary metabolite constellation for the detection of TCMR in renal allografts has been previously described by our group (7, 8). In combination with the eGFR values of patient, this test was previously proposed as a valuable support in biopsy decision-making and routine follow-up after renal transplantation. However, so far two main reasons limit the use of this test system in the clinic. The one is that all results generated so far were based on single center studies. The second reason is that the original cohort used to develop the test system was dominated by cellular rejections as these could be easily diagnosed in corresponding kidney biopsies at the time of development.

To overcome these limitations, the PARASOL study was initiated as a further completely independent validation study. Major aims of the PARASOL study were evaluation of the test system completely under routine conditions in six large, different European transplant centers. All centers were completely free in including suitable patients transplanted with donor organs retrieved as usually done in the respective country. Additionally, all treatment strategies, i.e., for immunosuppressive therapy, concomitant medication, routine follow-up visits, and renal biopsy, were on purpose not standardized to recruit a study population typical for renal transplant recipients all over Europe.

Successful recruitment of the study is described here along with an in-depth analysis of the study population, urine samples collected and correlation of these with biopsy findings, and analyses for the presence of donor specific antibodies in blood samples of the allograft recipients at the time of renal biopsies. All biopsies were evaluated by experienced renal pathologists according to the Banff classification systematics. Taking clinical findings, laboratory values, and histopathological results into account, the patients were grouped by the investigators into so called “cases,” “controls,” and “additional,” respectively. This will allow not only to discriminate rejecting from non-rejecting allografts. With this strategy, changes in the urine metabolome can be also correlated with pre-existing, i.e., donor-derived and *de novo* allograft damages over time and of all types, such as cellular and antibody mediated acute and chronic allograft lesions.

The complete documentation of 1,230 urine samples along with corresponding histopathology reports and blood analyses for donor specific antibodies from 972 different patients (approximately one third of them with rebiopsies during the study), will allow to evaluate the urinary metabolite changes for a wide variety of different allograft damages alone or in combination and with different severity, all being representative for typical patient courses after renal transplantation.

Future goals for the test progress are the validation of previous findings in an actual multicenter setting, detection and differentiating antibody-mediated rejection from cellular rejection and recognition of potential disruptive factors other causes of allograft damage.

In conclusion, the clinical part of the PARASOL study as an international, multicenter study representative for typical renal transplant patients in Europe could be finished successfully. All urine samples, renal biopsy pathology results, and biographic and clinical patient data are validated and now available for further spectroscopic and metabolomic analyses.

## Data Availability Statement

The raw data supporting the conclusions of this article will be made available by the authors, without undue reservation.

## Ethics Statement

The studies involving human participants were reviewed and approved by University Regensburg (principal investigator #16-315-101) and Local Ethics Committees. The patients/participants provided their written informed consent to participate in this study.

## Author Contributions

MB, GB, OV, LR, SN, ES, and BB designed the study. LG, OB, and H-JG gave conceptual advice to the study protocol and the enrollment strategy. MB, GB, OV, LR, LG, OB, and BB were local principal investigators for the participating transplant centers and enrolled together with their study personnel all participating patients. H-JG and KK gave advice on histopathological evaluation and classification of renal biopsy findings. MB, SN, VR, AS, TJ, CF, DZ, TB, FP, VH, and ES collected study material and data and were responsible for raw data controls. SN, VR, AS, and ES supervised and evaluated metabolomic analyses and performed statistical evaluations. MB and BB wrote the manuscript. All authors evaluated study data and contributed to the writing of the manuscript.

## Funding

This study was sponsored by numares AG.

## Conflict of Interest

SN, VR, AS, and ES report personal fees from numares AG, outside the submitted work. In addition, SN has a patent WO2018167157A1 pending that is directly related to this work. The remaining authors declare that the research was conducted in the absence of any commercial or financial relationships that could be construed as a potential conflict of interest.

## Publisher's Note

All claims expressed in this article are solely those of the authors and do not necessarily represent those of their affiliated organizations, or those of the publisher, the editors and the reviewers. Any product that may be evaluated in this article, or claim that may be made by its manufacturer, is not guaranteed or endorsed by the publisher.

## Footnotes

^1^ http://www.registrorenal.es/

## Abbreviations

ABMR: antibody-mediated rejection
AUC_ROC_: area under the ROC curve
CKD: chronic kidney disease
DSA: donor-specific anti-HLA IgG antibodies
eGFR: estimated glomerular filtration rate
NMR: nuclear magnetic resonance spectroscopy
ROC: receiver operating characteristic
TCMR: T-cell- mediated rejection
MFI: mean fluorescene intensity
MMF: mycofenolate mofetil
MPA: mycophenolic acid.
