# Supplementary material for: A Prospective Multicenter Trial to Evaluate Urinary Metabolomics for Non-invasive Detection of Renal Allograft Rejection (PARASOL): Study Protocol and Patient Recruitment
Source: Front Med (Lausanne). 2022 Jan 7;8:780585. doi: 10.3389/fmed.2021.780585 (PMC8782243; doi:10.3389/fmed.2021.780585)
Supplement: Supplementary file 1 [file Data_Sheet_1.docx]

**Table S1: Distribution of biopsy types shown for case, control and additional group.** For each of the three biopsy types, the total number of samples, as well as the number of samples within the three status groups are given. The relative frequencies are derived from the total numbers in the respective status group. Case group: Banff category 2 (ABMR) or 4 (TCMR), either alone or in combination with other findings (other non-rejection changes); Control group ((no rejection): Banff categories 1 (normal biopsy), 5I (mild interstitial fibrosis and atrophy (IFTA)); Additional group (Banff categories 3 (suspicious for TCMR), 5II (moderate IFTA), 5III (severe IFTA) or 6 (other non-rejection changes)).

|  |  | **Total** | **Event-driven** | **Follow-up** | **Protocol** |
| --- | --- | --- | --- | --- | --- |
| **Number of samples** |  | 1230 | 536 | 104 | 590 |
| **Status** | *Case* | 237/1230 (19.3%) | 149/237 (62.9%) | 31/237 (13.1%) | 57/237 (24.1%) |
|  | *Control* | 541/1230 (44.0%) | 125/541 (23.1%) | 30/541 (5.5%) | 386/541 (71.3%) |
|  | *Additional* | 452/1230 (36.7%) | 262/452 (58.0%) | 43/452 (9.5%) | 147/452 (32.5%) |

Table S2: **Distribution of the three status groups within the biopsy reason groups.** For each of the three status groups case, control and additional, the total number of samples, as well as the number of samples within the three biopsy reason groups are given. The relative frequencies are derived from the total numbers in the respective biopsy reason group. Case group: Banff category 2 (ABMR) or 4 (TCMR), either alone or in combination with other findings (other non-rejection changes); Control group ((no rejection): Banff categories 1 (normal biopsy), 5I (mild interstitial fibrosis and atrophy (IFTA)); Additional group (Banff categories 3 (suspicious for TCMR), 5II (moderate IFTA), 5III (severe IFTA) or 6 (other non-rejection changes)).

|  |  | **Total** | **Case** | **Control** | **Additional** |
| --- | --- | --- | --- | --- | --- |
| **Number of samples** |  | 1230 | 237 | 541 | 452 |
| **Biopsy Reason** | *Event-driven* | 536/1230  (43.6%) | 149/536 (27.8%) | 125/536 (23.3%) | 262/536 (48.9%) |
|  | *Follow-up* | 104/1230  (8.5%) | 31/104 (29.8%) | 30/104 (28.8%) | 43/104 (41.3%) |
|  | *Protocol* | 590/1230  (48.0%) | 57/590  (9.7%) | 386/590 (65.4%) | 147/590 (24.9%) |

**Table S3: Distribution of Banff categories for case, control and additional group**. Number of samples and relative frequencies refer to the total number of samples in the respective status group. The dashes do not mean that the data is missing, but that the respective Banff category is not expected in the status group and therefore does not appear. Case group: Banff category 2 (ABMR) or 4 (TCMR), either alone or in combination with other findings (other non-rejection changes); Control group ((no rejection): Banff categories 1 (normal biopsy), 5I (mild interstitial fibrosis and atrophy (IFTA)); Additional group (Banff categories 3 (suspicious for TCMR), 5II (moderate IFTA), 5III (severe IFTA) or 6 (other non-rejection changes)).

|  | **Total** | **Case** | **Control** | **Additional** |
| --- | --- | --- | --- | --- |
| **Number of**  **samples** | 1230 | 237 | 541 | 452 |
| Banff 2 | 153/1230  (12.4%) | 153/237 (64.6%) | - | - |
| Banff 2 + 4 | 12/1230  (1.0%) | 12/237  (5.1%) | - | - |
| Banff 4 | 72/1230  (5.9%) | 72/237  (30.4%) | - | - |
| Banff 1 | 344/1230  (28.0%) | - | 344/541 (63.6%) | - |
| Banff 5I | 197/1230  (16.0%) | - | 197/541 (36.4%) | - |
| Banff 3 | 65/1230  (5.3%) | - | - | 65/452  (14.4%) |
| Banff 5II, 5III | 123/1230  (10.0%) | - | - | 123/452 (27.2%) |
| Banff 6 | 77/1230  (6.3%) | - | - | 77/452  (17.0%) |
| other | 187/1230  (15.2%) | - | - | 187/452 (41.4%) |

**Table S4:** **Distribution of Banff categories across the different time spans after transplantation.** For each Banff category the number of samples for each time after transplantation category is given. Relative frequencies in total refer to the total sample number. Relative frequencies given for each Banff category refer to the total number of this combination. The order of the Banff categories is defined by the assignment to the status case, control or additional. Case group: Banff category 2 (ABMR) or 4 (TCMR), either alone or in combination with other findings (other non-rejection changes); Control group ((no rejection): Banff categories 1 (normal biopsy), 5I (mild interstitial fibrosis and atrophy (IFTA)); Additional group (Banff categories 3 (suspicious for TCMR), 5II (moderate IFTA), 5III (severe IFTA) or 6 (other non-rejection changes)).

|  | **Banff category** | **Total** | **≤ 6 months** | **]6,12] months** | **]1,4] years** | **> 4 years** |
| --- | --- | --- | --- | --- | --- | --- |
| **Number of samples** | *Total* | 1230 | 628 | 132 | 222 | 248 |
| **Status: Case** | *Banff 2* | 153/1230 (12.4%) | 29/153 (19.0%) | 12/153 (7.8%) | 48/153 (31.4%) | 64/153 (41.8%) |
|  | *Banff 2 + 4* | 12/1230 (1.0%) | 5/12  (41.7%) | 1/12  (8.3%) | 3/12  (25.0%) | 3/12  (25.0%) |
|  | *Banff 4* | 72/1230 (5.9%) | 35/72 (48.6%) | 10/72 (13.9%) | 18/72 (25.0%) | 9/72  (12.5%) |
| **Status: Control** | *Banff 1* | 344/1230 (28.0%) | 264/344 (76.7%) | 38/344 (11.0%) | 30/344 (8.7%) | 12/344 (3.5%) |
|  | *Banff 5I* | 197/1230 (16.0%) | 109/197 (55.3%) | 24/197 (12.2%) | 29/197 (14.7%) | 35/197 (17.8%) |
| **Status: Additional** | *Banff 3* | 65/1230 (5.3%) | 40/65 (61.5%) | 10/65 (15.4%) | 12/65 (18.5%) | 3/65  (4.6%) |
|  | *Banff 5II,III* | 123/1230 (10.0%) | 26/123 (21.1%) | 16/123 (13.0%) | 39/123 (31.7%) | 42/123 (34.1%) |
|  | *Banff 6* | 77/1230 (6.3%) | 30/77 (39.0%) | 7/77  (9.1%) | 16/77 (20.8%) | 24/77 (31.2%) |
|  | *other* | 187/1230 (15.2%) | 90/187 (48.1%) | 14/187 (7.5%) | 27/187 (14.4%) | 56/187 (29.9%) |


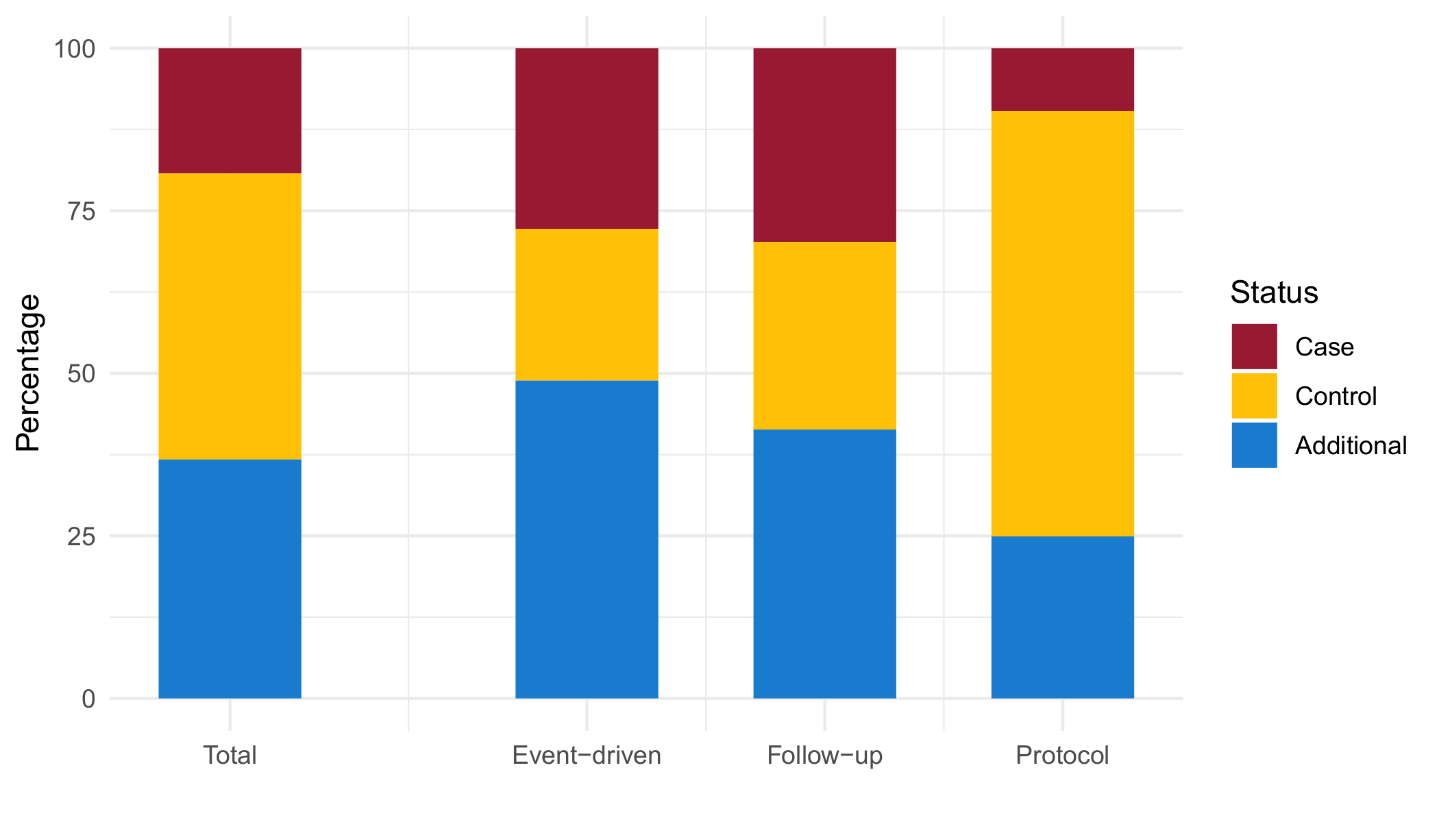


**Figure S1. Distribution of the status groups case, control and additional shown for each reason for biopsy.** Relative frequencies are displayed for the total number of samples (irrespective of the status assignment), as well as for each of the three biopsy reasons. Case group: Banff category 2 (ABMR) or 4 (TCMR), either alone or in combination with other findings (other non-rejection changes); Control group ((no rejection): Banff categories 1 (normal biopsy), 5I (mild interstitial fibrosis and atrophy (IFTA)); Additional group (Banff categories 3 (suspicious for TCMR), 5II (moderate IFTA), 5III (severe IFTA) or 6 (other non-rejection changes)).
